# Toward a visualized classifier for depression: characterization of hemodynamic patterns using time-domain fNIRS

**DOI:** 10.3389/fpsyt.2026.1724011

**Published:** 2026-03-03

**Authors:** Cyrus Su Hui Ho, Shujun Jing, Zhifei Li, Gabrielle Wann Nii Tay, Rachael Rui Qi Loh, Kenneth De Sheng Tong, Jinyuan Wang, Junyi Li, E Du, Nanguang Chen

**Affiliations:** 1 Department of Psychological Medicine, Yong Loo Lin School of Medicine, National University of Singapore, Singapore, Singapore; 2 Department of Psychological Medicine, National University Hospital, Singapore, Singapore; 3 Department of Biomedical Engineering, National University of Singapore, Singapore, Singapore; 4 National University of Singapore (Suzhou) Research Institute, Suzhou, China; 5 School of Microelectronics, Shenzhen Institute of Information Technology, Shenzhen, Guangdong, China

**Keywords:** brain activation, cerebral hemodynamics, depressive disorder, machine learning, time-domain fNIRS

## Abstract

**Background:**

Major depressive disorder (MDD) is a chronic illness associated with considerable morbidity and is characterized by high rates of recurrence and relapse. Early and accurate identification of depressive symptoms results in better treatment outcomes. However, the current diagnostic process relies mainly on subjective clinical interviews, underscoring the need for cost-effective physiological markers.

**Method:**

Increasing evidence suggests that alterations in neurovascular processes affect the cognitive and brain functions of individuals with MDD. This study introduced a time-domain functional near-infrared spectroscopy (TD-fNIRS) instrument and a test-retest protocol to characterize prefrontal hemodynamics in MDD. Utilizing a dataset of 27 patients with MDD and 27 age- and gender-matched healthy controls (HC), the study investigated differential hemodynamic patterns in the prefrontal cortex between MDD and HC through a visual analysis method, which included the separation of hemodynamic responses, feature extraction, and supervised classifiers.

**Result:**

A novel feature combination, the 'Integral and Centroid of Activation' derived from task-rest HbO ratio, was identified as the most effective optical biomarker in distinguishing MDD from controls. Utilizing only two features, the linear discriminant analysis attained average accuracies of 75.1% ± 6.6% across five-fold cross-validation.

**Conclusion:**

The results suggest that individuals with MDD exhibit a higher change in HbO relative to their initial HbO levels, indicating a greater oxygenation demand to support prefrontal cortex activation during speech and memory processes. This pilot study utilizing multichannel TD-fNIRS technology on human subjects provides new insights into replicable physiological features, potentially enabling objective measurement of the underlying neuropathological symptoms of MDD.

## 1 Introduction

Major depressive disorder (MDD) is a widespread condition with a significant global burden (1). Estimates indicate that depression affects approximately 5–13% of the population, meaning over 300 million individuals worldwide currently experience this disorder (2). Like many neuropsychiatric disorders, MDD is a complex syndrome characterized by varying symptoms and treatment responses. Common manifestations include persistent low mood, fatigue, cognitive difficulties such as memory impairment and poor concentration, disrupted sleep and appetite, diminished interest or pleasure, and feelings of guilt or worthlessness (3). In severe cases, depression can lead to psychosis, suicidal behavior, and an increased risk of premature death (4). It significantly hampers occupational functioning and negatively impacts overall quality of life (5).

Depression is currently diagnosed based on patients’ self-reported symptoms and an assessment of their clinical history over time. This approach relies heavily on physicians' expertise, which makes it susceptible to subjectivity and potential bias. Furthermore, psychiatric evaluations through clinical interviews and standardized questionnaires require administration by highly trained psychiatrists, increasing healthcare costs and potentially limiting access for some individuals. Therefore, incorporating objective, biomarker-based assessments could enhance early detection, improve treatment strategies, and alleviate the medical burden on both patients and clinicians.

Timely detection of major depressive disorder (MDD), followed by appropriate monitoring and treatment, is associated with higher remission rates, reduced functional impairment, and lower risk of chronicity (6, 7). The underlying mechanisms of depression are considered to be complex, involving interactions between neurobiological, genetic, and psychosocial factors (8). Growing evidence indicates that individuals with depression exhibit abnormalities in neurovascular processes. Recent clinical research (9) suggests that depression-related cognitive impairments are linked to pathology of the neurovascular unit (NVU). As a key interface regulating the exchange of substances between the brain and bloodstream, the NVU plays a crucial role in maintaining central nervous system homeostasis. Evidence from animal studies also suggests that the NVU influences cognitive function and overall brain activity (10). Furthermore, disruptions in neurovascular unit (NVU) function and blood–brain barrier integrity have been linked to depression-like behaviors in preclinical models and to major depressive disorder in clinical studies, although the precise mechanisms connecting these changes to mood and cognition remain incompletely understood (11, 12).

An emerging viewpoint in neuroscience suggests that abnormalities in the prefrontal cortical activity may underlie the pathophysiology of depressive disorder (13). Among various neuroimaging modalities, including functional magnetic resonance imaging (fMRI) and positron emission tomography (PET), functional near-infrared spectroscopy (fNIRS) stands out as a particularly suitable tool for investigating physiological markers in depression, due to its portability, high temporal resolution, cost-effectiveness, and suitability for real-world assessments despite its limitations in spatial resolution and penetration depth (1–2 cm). Consequently, an increasing number of psychiatric studies have utilized fNIRS to identify distinct neural patterns in depressed individuals compared to healthy controls (14, 15).

Although previous studies have demonstrated the utility of fNIRS in psychiatric research and identified altered prefrontal cortical activity as a potential indicator of MDD, the validation of fNIRS-based markers and the elucidation of the pathophysiology of depression remain limited due to several factors: (a) Insufficient evidence supports its accuracy and interpretability for symptom identification and individual-level diagnosis; (b) The widespread use of continuous-wave fNIRS (CW-fNIRS) instruments in recent research, which analyze changes in light intensity based on the Modified Beer-Lambert Law (16), is inherently affected by systemic artefacts (17) and has limitations in quantifying physiological parameters during the resting state (18); (c) Obtaining consistent inter- and intra-subject results remains challenging with CW-fNIRS, primarily due to its relatively low signal-to-noise ratio (SNR) and the variability of scattering coefficients among subjects (19, 20).

In addition, cerebral hemodynamic responses typically exhibit two distinct patterns: activation and suppression. The activation pattern is characterized by an increase in HbO following stimulus onset, which gradually returns to baseline during the post-task period, consistent with the expected neuronal response to events. By contrast, the suppressive pattern is marked by a decrease in HbO below baseline (zero) during the stimulus phase. Previous studies (17, 21) have suggested that such suppressive components may arise from extracerebral tissue or occur when participants experience mental stress during the task, leading to reduced blood flow and lower HbO levels. Current analytical approaches commonly classify channels into broad cortical regions of interest (ROIs), such as frontal or temporal areas, and rarely examine potential hemodynamic dysfunction by explicitly comparing these two response patterns. Consequently, the specific roles of activation versus suppression responses in the depression condition require further exploration.

Time-domain or time-resolved fNIRS technology utilizes ultrashort light pulses to illuminate biological tissue. The arrival times of photons, referred to as the ‘Temporal Point Spread Function (TPSF)’ or ‘Time-of-Flight (ToF) Distribution’, are recorded with picosecond or nanosecond resolution to assess tissue optical properties. These properties can be further analyzed to investigate the dynamics of hemoglobin and oxygenation levels within the microvasculature of the cerebral surface (22, 23). Preliminary studies have revealed that the falling edge of TPSF signals, primarily shaped by photons propagating through deeper tissue layers, is crucial for deriving physiological parameters within small vessels (24).

In this study, the patient group comprised individuals diagnosed with major depressive disorder. The terms "depression" and "MDD" are used interchangeably throughout the manuscript and **Supplementary Materials** to refer to this condition. Based on the widely used verbal fluency task (VFT), which is extensively employed in studies assessing cognitive deficits associated with depression (25), we introduce a multichannel TD-fNIRS instrument and a test-retest task protocol to investigate the prefrontal hemodynamic patterns in individuals with MDD. By combining baseline levels with task-induced changes in oxygen-hemoglobin, we aim to characterize the activated and suppressed responses involved in memory and language functions, which enables an objective analysis of the underlying neuropathological symptoms of MDD.

## 2 Methods

### 2.1 Participants and ethics

This study enrolled 27 patients diagnosed with depression [male/female: 12/15; mean age: 27.9; standard deviation (SD): 7.3] from the outpatient psychiatric clinics of a university hospital in Singapore, along with 27 healthy individuals (male/female: 9/18; mean age: 27.8; SD: 9.4), between April 2023 and January 2024. The diagnosis of major depressive disorder was confirmed by a psychiatrist according to the DSM-5 criteria (26). On the day of participation, depressive symptoms and psychosocial functioning were assessed using the 17-item Hamilton Depression Rating Scale (HAM-D) (27). Healthy control participants were recruited from the community and screened to have no history of psychiatric disorders, as verified by a psychiatrist and confirmed by a medical history questionnaire.

All participants provided written informed consent. The study followed the ethical guidelines established in the Declaration of Helsinki and the Belmont Report. Ethical approval was obtained from the Domain Specific Review Board of the National Healthcare Group in Singapore (protocol number 2022/00164) and the Institutional Review Board of the National University of Singapore (reference number NUS-IRB-2022-259).

### 2.2 Analysis of sample characteristics and hemodynamic patterns

#### 2.2.1 Demographic and clinical data

Statistical analyses compared continuous and categorical variables between depressed patients and healthy controls (HC). Continuous variables (e.g., age, HAM-D scores) were analyzed using two-sample independent t-tests, while categorical variables (e.g., education level, smoking history) were assessed with Pearson chi-square tests. IBM SPSS Statistics 21.0 was used for all analyses; all tests were two-tailed, with a significance level set at p < 0.05.

#### 2.2.2 Hemodynamic parameters

To quantify the sensitivity of early and late measurement points in TPSF signals to the distinctive hemodynamic patterns of MDD, we compared activation, suppression and channel-averaging responses between the patient group and the control group by using two parameters representing the relative changes in oxygenated hemoglobin (HbO) concentrations ( $\Delta_{HbO}^{i}$, where *i* is the time delay, *i = 1, 2 or 3*) and the task-rest HbO ratio ( $R_{HbO}^{i}$, *i* is the delay pair, *i = 1 or 2*). They were all derived from the time-domain optical signal at different time delays (see Supplementary for the definition and more details). Furthermore, two feature variables of each hemodynamic response were extracted to characterize individual time-series samples: the integral value of the response, representing the intensity of brain activity over the task duration by integrating signal changes, and the centroid value, indicating the timing of the responses (28, 29). In **Supplementary Methods**, the calculation of HbO levels is provided in Sec.3, while the data processing and parameters extraction are detailed in Sec.4 and **Supplementary Figures S5**, **S6**.

#### 2.2.3 Channel rejection

Across all 108 trials (54 participants × 2 VFT sessions), an average of 7.5 ± 4.1 channels per 22-channel recording (34% ± 19%) were excluded due to corruption. The most frequently corrupted channels (1, 4, 5, 9, and 16) were primarily distributed in the upper-left and upper-right corners, largely due to poor optode-scalp coupling (**Supplementary Figure S9**). Our analytical approach, which characterizes generalized activated and suppressive responses rather than relying on specific channel signals, ensured that hemodynamic analysis supported valid group-level statistical inference.

#### 2.2.4 Feature selection

We performed statistical comparisons of hemodynamic parameters between HC and MDD groups using two-tailed t-test with a 95% confidence level. The test variables were the integral and centroid values of $\Delta_{HbO}^{i}$ and $R_{HbO}^{i}$ for hemodynamic responses to the VFT task. By reducing the multi-channel time-series HbO data to a compact set of features, we aimed to isolate the most discriminative hemodynamic patterns that distinguish depressed patients from healthy controls.

#### 2.2.5 Group classification

To further assess the ability of TD-fNIRS indices to independently discriminate the presence of depression, we evaluated classification performance on the selected features using conventional machine learning models. These methods and their parameters have been described in our previous work (30). In brief, we applied support vector machines (SVM) with linear kernel function, linear discriminant analysis (DA), decision trees (Dtrees) with maximum 3 splits, and naive Bayes (NB) with normal distribution assumption. Performance was quantified using classification accuracy and five-fold cross-validation. **Supplementary Figure S6** illustrates the data analysis pipeline, which encompasses data transformation, feature selection, and visualized classification models. The implementation code has been open-sourced to provide a transparent framework for future research and validation.

## 3 Results

### 3.1 Sample characteristics

Depressed patients and healthy controls (HC) did not differ significantly in age, sex, ethnicity, marital status, or history of drinking and smoking (p >.05; **Table 1**). However, there were significant differences in education (years) and HAM-D scores. Depressed patients had lower education levels compared to HC (χ² (1, N = 54) = 17.444, p <.001). As anticipated, depressed patients showed significantly higher HAM-D scores (t = 8.086, p <.001) and generated fewer words (t = 2.408, p = .020) compared to HC during the VFT task.

**Table 1:** Demographic characteristics of people with major depressive disorder and healthy controls

Demographics and Clinical Data	MDD (n = 27)	HC (n = 27)	P-value
Age(years)	27.89 (SD = 7.3)	27.81(SD = 9.4)	0.974
Sex			0.402
Male	12 (22.20%)	9 (16.70%)	
Female	15 (27.80%)	18 (33.30%)	
Ethnicity			0.715
Chinese	22 (40.70%)	23 (42.60%)	
Others	5 (9.30%)	4 (7.40%)	
Marital status			0.600
Single	24 (44.40%)	25 (46.30%)	
Married	2 (3.70%)	2 (3.70%)	
Divorced/Separated	1 (1.90%)	0 (0.00%)	
Education (years)			<0.001*
>16 years	12 (22.20%)	26 (48.10%)	
12–16 years	13 (24.10%)	1 (1.90%)	
< 12 years	2 (3.70%)	0 (0.00%)	
Smoking history	5 (9.26%)	1 (1.85%)	0.083
Alcohol history	14 (25.93%)	7 (12.96%)	0.051
Medication use	22/27 (81.5%)		
SSRI	14 (51.9%)		
NDRI	2 (7.4%)		
SNRI	3 (11.1%)		
Other	3 (11.1%)		
HAM-D	16.08 (SD = 7.10)	3 (SD = 2.51)	<0.001*
Mild (8-16)	9 (17.00%)	1 (1.90%)	
Moderate (17-23)	10 (18.90%)	0 (0.00%)	
Severe(≥24)	3 (5.70%)	0 (0.00%)	
Words Number	18.28 (SD = 5.40)	21.46 (SD = 5.64)	0.020*

*p-values ≤.05 are in bold.

HC, Healthy controls;; MDD: People with major depressive disorder.

SSRI, Selective Serotonin Reuptake Inhibitors.

NDRI, Norepinephrine-Dopamine Reuptake Inhibitors.

SNRI, Serotonin-Norepinephrine Reuptake Inhibitors.

The clinical MDD group (n=27) had a mean age of 27.9 years (55.6% female) and mean HAM-D score of 16.1, indicating moderate depression severity. Comorbidities were present in 5/27 patients (18.5%) and heterogeneous: anxiety disorders (n=2), respiratory conditions (n=3), hormonal disorder (n=1), and vestibular migraine (n=1). This lack of consistent pattern suggests minimal impact on group comparisons. Medication use was reported by 22/27 participants (81.5%), reflecting varied types, dosages, and treatment durations consistent with real-world clinical practice. The primary analysis compared MDD versus control groups rather than stratifying by medication status. Given this between-group design and high antidepressant prevalence, medication effects are unlikely to systematically confound the reported hemodynamic differences.

### 3.2 Statistical analysis of hemodynamic responses during the task

**Figure 1:**
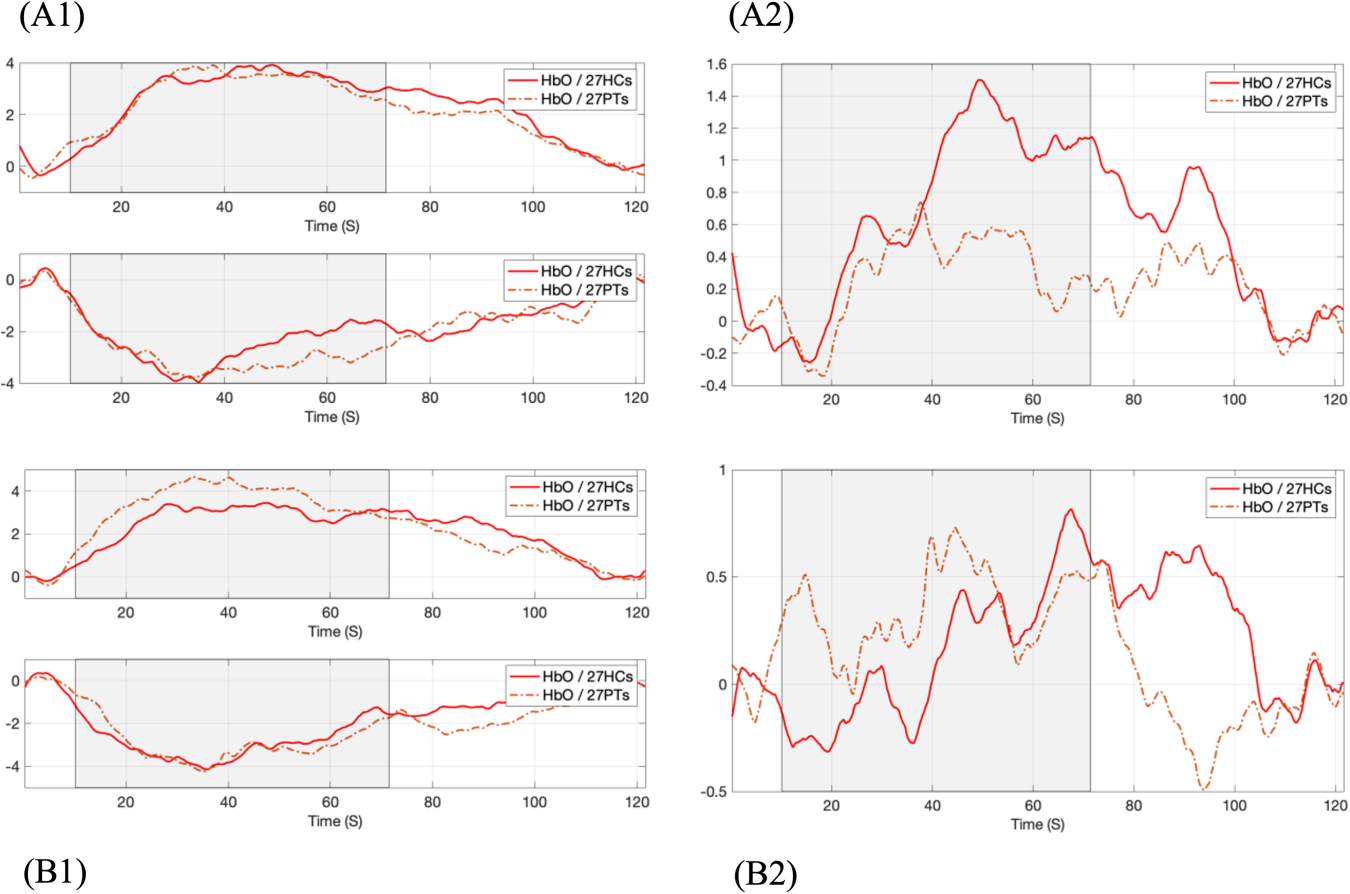
Comparison of average oxy-hemoglobin responses between HC and MDD during VFT task (gray area). The task-rest HbO ratio **(a)** $R_{HbO}^{1}$ and **(b)** $R_{HbO}^{2}$ derived with different delay pairs. (a1) and (b1) on left: activated and suppressive responses; (a2) and (b2) on right: channel-wise averaging response.

Two optically available hemodynamic response parameters were defined and used to differentiate MDD and HC groups, i.e., $\Delta_{HbO}^{i}$ and $R_{HbO}^{i}$, which were described in the **Supplementary Methods** Sec.3. It was found that the task-rest HbO ratio ( $R_{HbO}^{i}$) led to better classification results. Hemodynamic Reponses presented in **Figure 1** were based on this dimensionless parameter. In **Figure 1**(a1), the MDD group demonstrated a more pronounced suppressive response than the HC group (p-value = 0.05; **Table 2**). Moreover, **Figure 1**(a2) shows that the channel-averaging $R_{HbO}^{1}$ response increased less and activated earlier (p-value = 0.04; **Table 2**) in MDD compared to HC during the task period.

**Table 2:** P-value (* ≤ 0.05) of features from relative HbO change ( $\Delta_{HbO}^{i}$) and task-rest HbO ratio ( $R_{HbO}^{i}$) during the verbal fluency task, comparing people with MDD and healthy controls in statistical analysis.

Oxyhemoglobin Data	Time Delay	Activation		Suppression		Channel-averaging	
		Integral	Centroid	Integral	Centroid	Integral	Centroid
$\Delta_{HbO}^{i}$	Delay 1	0.81	0.19	0.1	0.18	0.59	0.01^*^
	Delay 2	0.65	0.05^*^	0.04^*^	0.52	0.46	0.05^*^
	Delay 3	0.81	0.11	0.29	0.16	0.58	0.22
$R_{HbO}^{i}$	Delay 1-2	0.6	0.41	0.05^*^	0.1	0.2	0.04^*^
	Delay 2-3	0.0018^*^	0.03^*^	0.9	0.52	0.51	0.03^*^

$\Delta_{HbO}^{i}$, where i was the time delay index. This parameter was derived from the time-domain signal at the ith time delay using the Modified Beer-Lambert Law (MBLL). $R_{HbO}^{i}$, the ratio of the task HbO concentration to the rest state concentration derived from the Diffusion Equation (DE) model and TD-fNIRS measurements.

*p-values <.05 are in bold. For the < symbol, use the symbol that represents less than or equal.

Analysis of $R_{HbO}^{2}$ data derived from later-arriving TPSF signals revealed a stronger and earlier activated response in the MDD group compared to controls (p-values = 0.0018, 0.03; **Table 2**), while both groups showed similar changes in amplitude and timing for the suppressive response, as illustrated in **Figure 1**(b1). For the channel-averaging response (**Figure 1**(b2)), the centroid value of $R_{HbO}^{2}$ indicated that the response timing of MDD was significantly earlier than that of HC (p-value = 0.03; **Table 2**), following the pattern of the activated response.

### 3.3 Distinguish people with depression from controls by hemodynamic responses

As several variables of hemodynamic responses from the $\Delta_{HbO}^{i}$ or $R_{HbO}^{i}$ data indicated significant differences between depression and control groups (**Table 2**), these indicators were extracted to represent smulti-dimensional features for each sample and applied in common machine learning models to test their classification performance. Due to limited space and the obvious superior performance of the proposed task-rest HbO ratio ( $R_{HbO}^{i}$) over the relative HbO change ( $\Delta_{HbO}^{i}$), we present results from the $R_{HbO}^{i}$ features in this section, while the results related to the conventional $\Delta_{HbO}^{i}$ features are included in the **Supplementary Results** Sec.1.

Based on the statistical differences in **Table 2**, five features derived from the $R_{HbO}^{i}$ data were identified as the hemodynamic patterns that could differentiate individuals with MDD from HC. The group-level comparisons of these discriminative features between two groups are plotted on a common scale in **Supplementary Figure S8**, wherein two features correspond to integral values and three features correspond to centroid values. **Supplementary Figure S8** (a) and (c) show that the change amplitudes of $R_{HbO}^{i}$ from the MDD group were higher than the HC group in both suppressive and activated responses. **Supplementary Figure S8** (b), (d), and (e) demonstrate that the response timing in MDD was consistently earlier than in HC.

Since five features from the task-rest HbO ratio ( $R_{HbO}^{i}$) data showed statistical differences between HC and MDD, we selected all these (5D) features to test overall performance and used only two-dimensional (2D) features to construct a visualized classifier. Among the feature sets, five preferred feature combinations and their performance for identifying MDD cases are demonstrated in **Table 3**, which presents the discriminant accuracy and the five-fold cross-validation results, respectively. **Table 3** reveals that the 5D features differentiated MDD cases from controls with superior performance across all classification models. Specifically, the NB classifier achieved an accuracy of 82.4%, with a sensitivity of 75.9% (true positive = 41 out of 54 MDD samples) and a specificity of 88.9% (true negative = 48 out of 54 control samples). Moreover, the five-fold cross-validation results in **Table 3** indicate that when cooperating with two groups of 2D features (i.e., F7 & F8 and F7 & F12), the DA model achieved comparable and more stable prediction rates. With fewer features, the model avoided overfitting, resulting in average accuracies of 75.1% ± 6.6% and 73.2% ± 3.7% across the five dataset splits.

**Table 3:** Classification accuracy and five-fold cross-validation accuracy (Mean ± Std.) of 2D and 5D features extracted from the task-rest HbO ratio ( $R_{HbO}^{i}$).

Classifier	F3 & F7	F6 & F7	F7 & F8	F7 & F12	5D Features
Linear SVM	66.70%	68.50%	74.10%	74.10%	77.80%
	66.7% ± 4.9%	66.8% ± 12.3%	73.2% ± 5.8%	68.7% ± 9.4%	71.3% ± 8.1%
Discriminant Analysis	63.00%	66.70%	73.10%	74.10%	78.70%
	63.9% ± 9.9%	69.5% ± 10.7%	75.1% ± 6.6%	73.2% ± 3.7%	78.7% ± 12.5%
Decision Trees	71.30%	72.20%	72.20%	72.20%	77.80%
	65.8% ± 7.0%	64.8% ± 8.3%	63.9% ± 6.5%	60.2% ± 6.9%	62.0% ± 15.0%
Naive Bayes	70.40%	71.30%	75.00%	72.20%	82.40%
	61.1% ± 7.2%	66.7% ± 11.6%	62.9% ± 13.3%	63.9% ± 7.4%	74.8% ± 8.9%

F3: Integral of suppressive $R_{HbO}^{1}$.

F6: Centroid of channel-averaging $R_{HbO}^{1}$.

F7: Integral of activated $R_{HbO}^{2}$.

F8: Centroid of activated $R_{HbO}^{2}$.

F12: Centroid of channel-averaging $R_{HbO}^{2}$.

5D Features: F3, F6, F7, F8, and F12.

**Figure 2:**
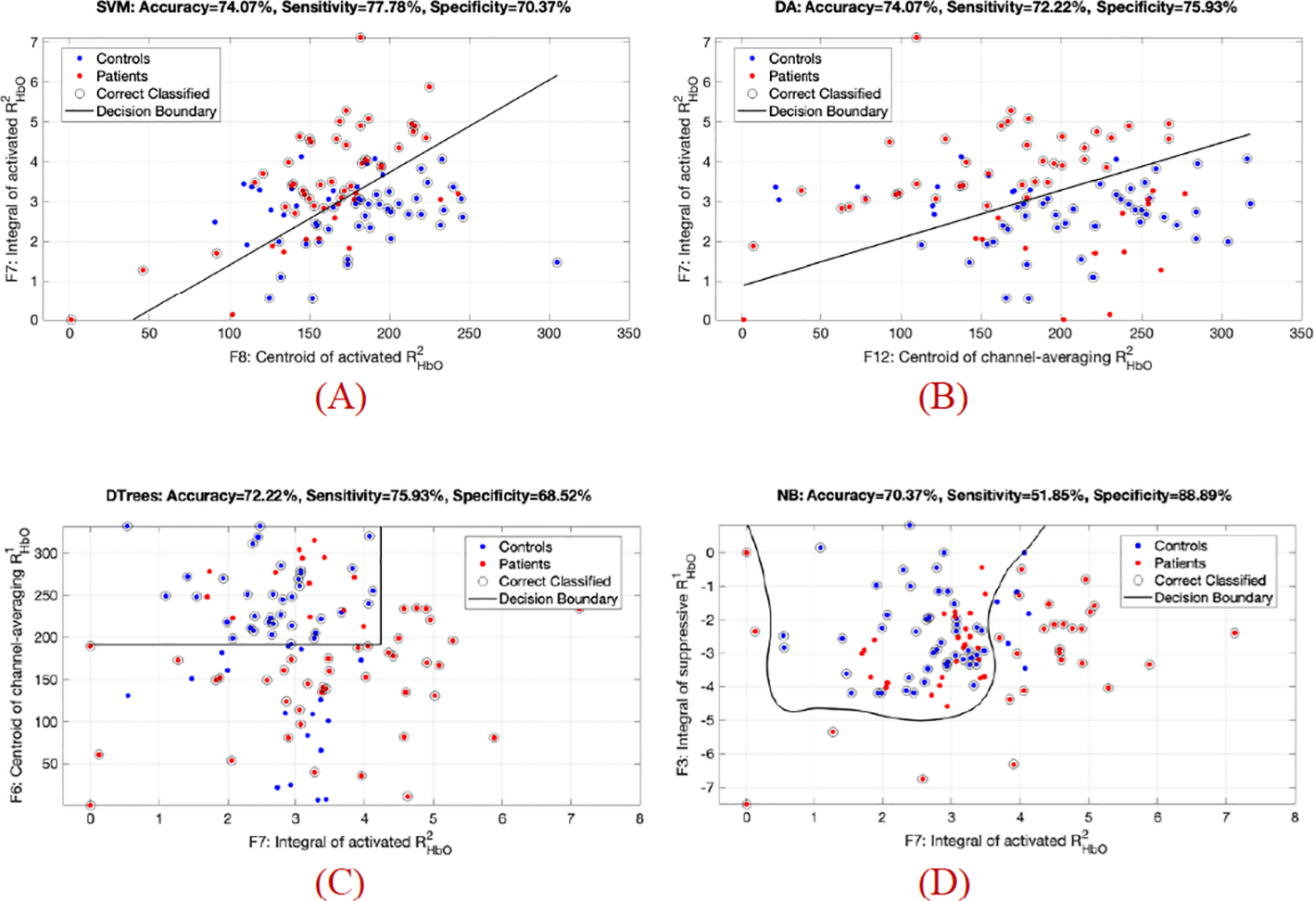
Scatter plot with the 2D features of task-rest HbO ratio ( $R_{HbO}^{i}$) for the patient (red) and control (blue) groups; the decision boundary was determined by the machine learning classifier. **(A)** SVM classifier using F8/F7; **(B)** DA classifier using F12/F7; **(C)** DTrees classifier using F7/F6; **(D)** NB classifier using F7/F3.F7/F3.

Using the 2D features and a decision boundary determined by the machine learning model, four representative scatter plots for classifying each sample are illustrated in **Figure 2**. For example, **Figure 2** (b) shows that the DA model achieved an overall accuracy of 74.1%, with a sensitivity of 72.2% (true positive = 39 out of 54 MDD samples) and a specificity of 75.9% (true negative = 41 out of 54 control samples). Full results for all 2D feature combinations of $R_{HbO}^{i}$ responses can be found in the **Supplementary Tables S3**, **S4**.

Additionally, the classification performance of features obtained from the relative HbO change ( $\Delta_{HbO}^{i}$) was tested using the same processing methods and classifiers. Firstly, the time-series $\Delta_{HbO}^{i}$ obtained by Modified Beer-Lambert Law (MBLL) have shown similar response patterns for the three TPSF delays, as illustrated in the **Supplementary Figure S7**. Specifically, **Supplementary Figure S7** (b1)-(b2) compares the mean $\Delta_{HbO}^{2}$ derived from the 2^nd^ TPSF delay. A stronger suppressive response was observed in the MDD group relative to the HC group (p-value = 0.04; **Table 2**), while the amplitudes in $\Delta_{HbO}^{2}$ shows no significant difference for the activated response. The stronger suppression, leading to a lower channel-averaging $\Delta_{HbO}^{2}$, was observed in MDD compared to HC and was consistent with common findings from previous studies using CW-fNIRS and MBLL analysis during the VFT task for depression (29, 30). Secondly, as shown in **Supplementary Tables S1**, **S2**, no combination of 2D or more features from $\Delta_{HbO}^{i}$ matched the performance of features from the task-rest HbO ratio ( $R_{HbO}^{i}$) in terms of both prediction accuracy and five-fold cross-validation. These preliminary results suggest that the proposed $R_{HbO}^{i}$ response is more sensitive in reflecting the distinctive hemodynamic patterns of MDD compared to the conventional $\Delta_{HbO}^{i}$ response.

## 4 Discussion

The pathophysiology of major depressive disorder (MDD) involves distinct alterations in cerebral function affecting emotion regulation and reward processing. Prior research suggests that a key feature of this dysregulation may be an imbalance in prefrontal activity: under-activity in regions like the dorsolateral prefrontal cortex alongside over-activity in others such as the orbitofrontal cortex, which is implicated in reward and loss processing relevant to MDD (31, 32). However, accurately identifying the distinctive patterns of depression from complex and noisy brain signals remains a challenge in clinical practice. In this work, we utilized a 22-channel time-domain fNIRS instrument to collect hemodynamic time-series data during a two-session verbal fluency task, resulting in a dataset of 54 depression samples (27 patients × 2 trials) and 54 healthy samples (27 controls × 2 trials). Subsequently, we investigated the differential hemodynamic patterns of the prefrontal cortex between MDD patients and healthy controls (HC) using a visualized discriminant analysis method, which includes separating hemodynamic responses, feature extraction of oxygenated hemoglobin (HbO), and supervised pattern recognition models.

This study firstly examined cognitive performance and hemodynamic responses during a verbal fluency task across two repeated sessions. No significant differences were found between sessions for either task performance (word count) or HbO-derived features, as assessed by the two-tailed t-test. This result replicates prior findings on the test-retest reliability of multi-session fNIRS measurements during the verbal fluency task (33, 34). Secondly, in line with earlier fMRI and fNIRS studies (34–36), our TD-fNIRS measurements confirmed typical prefrontal hemodynamic responses induced by a cognitive task. The conventional Modified Beer-Lambert Law (MBLL) analysis also revealed lower channel-averaged HbO changes ( $\Delta_{HbO}^{i}$) in MDD participants compared to healthy controls.

Furthermore, we identified five features from the task-rest HbO ratio ( $R_{HbO}^{i}$) that showed significant differences between MDD and controls. Specifically, consistent with prior research (30), three features obtained from centroid variables indicated that individuals with MDD exhibited a premature decline in HbO response and may have difficulty sustaining activation compared to healthy controls. Two additional significant features calculated using integral variables, which represent the change amplitudes of suppressive and activated task-rest HbO ( $R_{HbO}^{i}$) responses, were found to be stronger in the depression group than in the control group. Notably, the feature 'Integral of Activation at delay pair 2–3 of time-domain signal' was the most effective in differentiating MDD from controls (see **Supplementary Tables S3**, **S4**).

Through separating the hemodynamic responses into activation and suppression, then focusing on the activation component rather than a conventional region-of-interest analysis, our results suggest that individuals with depression exhibit a higher proportion of change in HbO relative to the initial HbO level, indicating a greater oxygenation demand to support prefrontal activation involved in speech and memory processes. This observation can be interpreted within several converging frameworks of depression neuropathology. First, it supports the cognitive effort hypothesis, wherein compensatory hyperactivation reflects greater neural effort to overcome underlying inefficiencies (37, 38). Second, it relates to evidence of neurovascular and metabolic dysregulation in depression, where altered cerebral blood flow and energy metabolism may necessitate a proportionally larger hemodynamic response to meet neural oxygen demands (39, 40). Finally, our TD-fNIRS metric extends these models by suggesting that, when activation does occur, it may capture a novel hemodynamic signature of inefficient prefrontal recruitment and heightened metabolic cost of cognitive engagement in depression.

As shown in the **Supplementary Tables S1**-**S4**, the full prediction results of all combinations with the identified significant features indicate that the classification performance using features from task-rest HbO ( $R_{HbO}^{i}$) were substantially superior to that using features from relative HbO change ( $\Delta_{HbO}^{i}$) generated by MBLL in common fNIRS studies. These results imply task-rest HbO ( $R_{HbO}^{i}$) signals may have preferable characteristics that contribute to identifying the distinctive hemodynamic patterns of MDD, including:

1. TD-fNIRS measurements enable the separation of activated and suppressive responses along with depth-relevant information, represented by early and late time-of-arrival photons in time-domain signals.
2. The time-series task-rest HbO ( $R_{HbO}^{i}$) represent relative changes in HbO levels via the ratio of task and rest state oxyhemoglobin concentrations, a feature unique to TD-fNIRS systems. This approach may enhance detection of stimulus-induced brain activity by partially mitigating effects of individual variability in systemic blood hemoglobin and tissue scattering properties, thereby facilitating more standardized comparisons across subjects or monitoring conditions (41).

The classification map using only two-dimensional features allows for the adoption of simple models, such as a linear support vector machine, to achieve robust classification of small-scale datasets. Moreover, unlike results from complicated 'black box' analyses, this objective and visualizable evaluation of MDD patients using the simplest and fewest variables provides clear benefits for generating diagnostic reports and tracking disease progress in psychiatric practice.

On the other hand, this study has several limitations. The first is the small sample size, which prevents a comprehensive examination of the identified features in relation to diverse confounding factors, such as demographic and clinical variables. Furthermore, the relationship between TD-fNIRS measures and individual HAM-D items, including suicidal ideation, could not be established. Another methodological limitation is the disparity in years of education among the groups, a factor associated with verbal fluency that may have confounded the hemodynamic measures. This underscores the need for future studies involving larger, education-matched, or longitudinal cohorts. Additionally, while including more significant features in the classifier enhances performance metrics, it also requires more data to minimize variance across folds during cross-validation.

Secondly, due to the limited spatial and depth resolution of fNIRS technology, it remains unclear how neurovascular processes induce activation and suppression patterns and whether these occur in specific regions of the prefrontal cortex. Since later delays represent longer optical path lengths of photons in time-domain signals for TD-fNIRS measurements, the relationship between time delays of signals and the detection depth of brain tissue could be established and verified by combining TD-fNIRS with fMRI in future studies. This would also help clarify the sensitivity and depth of fNIRS measurements in detecting cerebral hemodynamic dysfunction.

Thirdly, channel corruption (34% ± 19%) reduced spatial coverage, reflecting typical fNIRS challenges in verbal tasks. Future work should optimize headgear and motion correction for improved data quality. Lastly, the analysis of integral and centroid variables for averaging responses may oversimplify the hemodynamic characteristics of cerebral activity. A larger sample size would allow the use of multimodal data mining and feature selection algorithms to analyze multichannel time-series signals, potentially identifying more reliable markers to assess the heterogeneous syndrome in depression.

Despite these constraints, this work provides insights into hemodynamic dysfunctions in people with depression through time-domain fNIRS measurements. With rapid advances in TD-fNIRS instrumentation, this technology shows promise as a potential adjunct to clinical evaluation, enabling real-time, sensitive monitoring of cerebral hemodynamic changes and quantification of hemoglobin concentration in a portable system. These developments may provide clinicians with additional quantifiable hemodynamic information to support depression diagnosis and investigation of associated neuropathological processes.

## 5 Conclusions

Cortical oxygen-hemoglobin responses measured by an optical topography system serve as a direct and sensitive indicator of cerebral neurophysiological function. This study introduced a TD-fNIRS instrument along with a test-retest paradigm to identify varying patterns of depression from cerebral activation and suppression responses. The statistical and classification results revealed greater task-rest HbO ratio increases and earlier onset of prefrontal cortex activation in individuals with MDD, indicating a distinctive cerebral hemodynamic pattern associated with this disorder across multiple verbal fluency task sessions.

Using simple classification models and a limited set of predictors, our findings suggest TD-fNIRS measurements could serve as an objective adjunct to clinical assessment, offering continuous hemodynamic monitoring with evaluation outputs readily interpretable by clinicians to support depression management decisions. Future research utilizing large-scale TD-fNIRS datasets may enable more robust, quantifiable assessment of depressive symptoms alongside traditional clinical evaluation.

## Data Availability

The raw data supporting the conclusions of this article will be made available by the authors, without undue reservation. The MATLAB code in this study are available at https://www.mathworks.com/matlabcentral/fileexchange/180528-characterizing-hemodynamic-pattern-in-depressionby-td-fnirs.
