# Supplementary material for: Toward a visualized classifier for depression: characterization of hemodynamic patterns using time-domain fNIRS
Source: Front Psychiatry. 2026 Mar 3;17:1724011. doi: 10.3389/fpsyt.2026.1724011 (PMC12992268; doi:10.3389/fpsyt.2026.1724011)
Supplement: Supplementary file 1 [file SupplementaryFile1.docx]

**Supplementary**

**List of Abbreviations and Acronyms:**

| **Abbreviations** | **Definitions** | **Abbreviations** | **Definitions** |
| --- | --- | --- | --- |
| MDD | Major depressive disorder | TD-fNIRS | Time-domain functional near-infrared spectroscopy |
| HC | Healthy controls | fMRI | Functional magnetic resonance imaging |
| MBLL | Modified Beer-Lambert Law | PET | Positron emission tomography |
| VFT | Verbal fluency task | CW-fNIRS | Continuous-wave functional near-infrared spectroscopy |
| SD | Standard deviation | TPSF | Temporal Point Spread Function |
| HAM-D | Hamilton Depression Rating Scale | ToF | Time-of-Flight Distribution |
| PFC | Prefrontal cortex | VCSEL | Vertical cavity surface-emitting laser |
| Hb | Hemoglobin |  |  |
| HbO | Oxygenated hemoglobin | DE | Diffusion equation |
| HbR | Deoxygenated hemoglobin | SVM | Linear support vector machine |
| HbT | Total Hemoglobin | DA | Discriminant analysis |
| BOLD | Blood oxygenation level-dependent | Dtrees | Decision trees |
| $\Delta_{HbO}^{i}$ | Relative change of oxygenated hemoglobin | NB | naive Bayes |
| $R_{HbO}^{i}$ | Task-rest ratio of oxygenated hemoglobin | NVU | Neurovascular unit |

**Methods:**

1. **Experimental paradigms**

A well-established and easy-to-operate paradigm, the verbal fluency task (VFT) was selected to assess cognitive abilities, including speech and memory functions. Previous studies demonstrated that the VFT elicits consistent brain activation patterns [1][2]. This is evidenced by a high correlation in hemodynamic waveforms (mean Pearson's correlation coefficient 0.77 ± 0.21) and the replication of stable frontal activation and task performance across repeated sessions. Therefore, the VFT is a reliable and reproducible paradigm for fNIRS studies, making it suitable for psychological and neurocognitive assessments of psychiatric disorders [3].


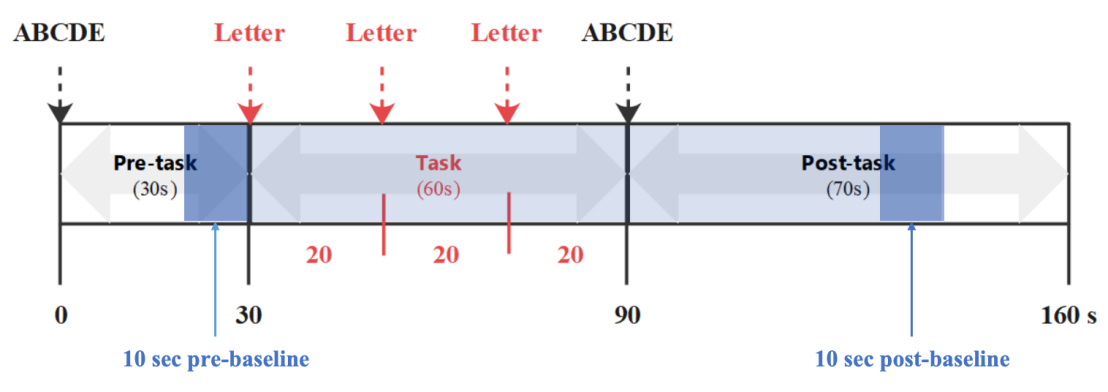


1. Design of verbal fluency task

The paradigm design of the VFT is illustrated in Figure S1. The 160-second block-design VFT comprises three periods: a 30-second pre-task, a 60-second task period, and a 70-second post-task period. For the pre- and post-task baseline periods, the subject was instructed to slowly and consecutively repeat five letters (i.e., A, B, C, D, and E) aloud. During the task period, the participant was instructed to generate as many English words starting with a designated letter as possible, with each letter changing every 20 seconds throughout the 60-second task; the stimulus letters were randomly selected and differed between the practice session and the two task trials. The subject was required to repeat the five letters instead of remaining silent during the pre-task and post-task periods. This procedure allows us to differentiate between the word-generating and utterance processes [4]. The contrast between the vocalization and verbal fluency conditions was utilized to enhance the specificity of the verbal fluency readout from the TD-fNIRS system.

1. **Time-domain functional NIRS measurements**

At the start of the study, participants were seated in front of a computer screen during the scan (Figure S2 (a)) and instructed to minimize eye blinking, biting, and head movement to avoid artifacts. To confirm their understanding of the instructions, they first practised the task without the head cap and received feedback from the research staff. To examine the replicable hemodynamic pattern of depression during the same cognitive stimulation across multiple trials, the VFT was performed in two sessions with a 10-minute interval between them. Consequently, data from 27 patients (2 trials each = 54 depression samples) and 27 controls (2 trials each = 54 healthy samples) were collected for discriminant analysis.


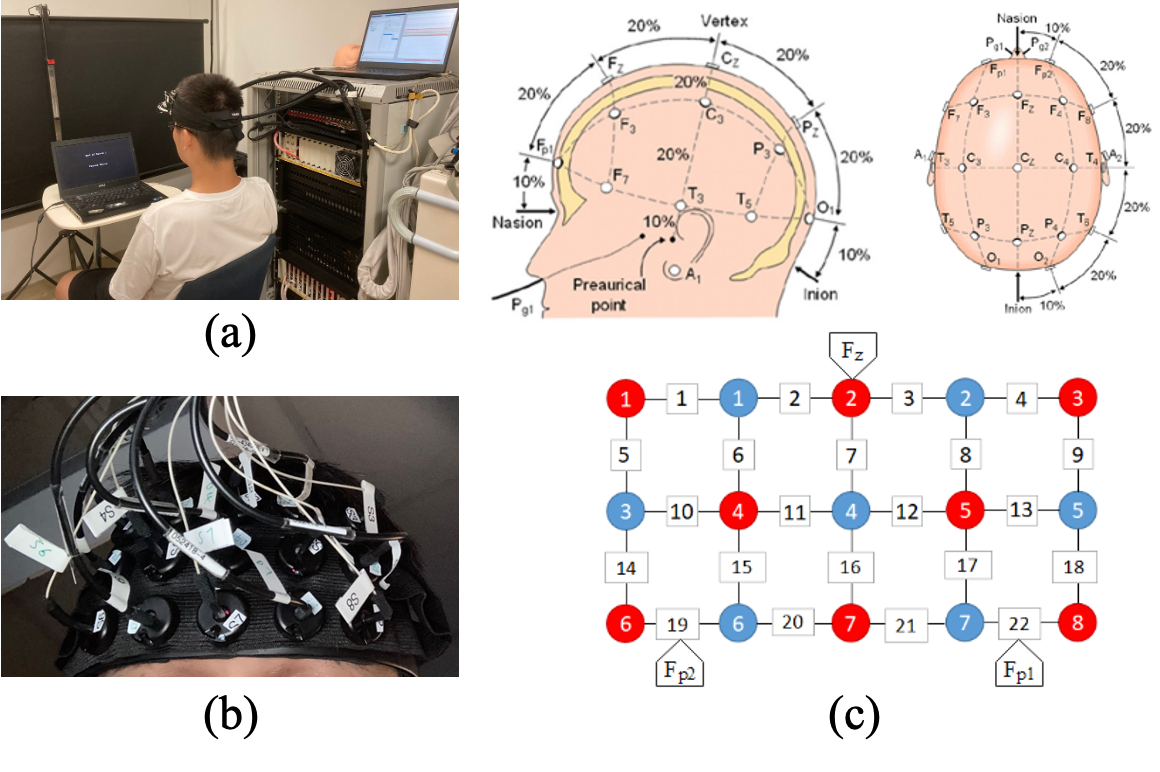


1. (a) Measuring prefrontal hemodynamics while the subject performs psychological tasks. (b) Optical topographic image of the frontal head. (c) Channels localization: red and blue circles indicate the locations of NIR light sources and detectors respectively, while the rectangles represent probing areas, i.e., channels.

We previously reported a novel implementation of a TD-fNIRS system that utilized a vertical cavity surface-emitting laser (VCSEL) diode modulated with pseudo-random bit sequences, recovering TPSF measurements through demodulation circuits [5]-[7]. The system has recently been upgraded to include dual wavelengths (680 nm and 850 nm), along with eight light sources and seven detectors, allowing for simultaneous 22-channel measurements at a sampling rate of 5.5 Hz. As shown in Figure S2 (b), the 22-channel headcap covered the frontal region, measuring hemodynamic parameters from the prefrontal cortex (PFC). The setup consisted of eight sources and seven detectors mounted in a probe holder arranged in a 3 × 5 grid. Each source-detector pair was spaced 2.5 cm apart. To ensure optimal coverage of the PFC, the probe array was positioned in the frontal region according to the international 10-20 system [8]. Specifically, source-2 was aligned with Fz, while the midpoints of channels 19 and 22 were located at Fp2 and Fp1, respectively (Figure S2 (c)).

Figure S3 shows a sample of 22-channel TPSF signals at two wavelengths during the resting state. For the channel at each source-detector pair, the TPSF signal is denoted as $S_{k}^{\lambda}=C\Phi^{\lambda}\left( kT_{0} \right)$. Here $\lambda$ is the source wavelength, *k* is the time delay, $T_{0}=0.4 ns$ is the time resolution, $\Phi$ is the time-dependent fluence rate, and *C* is a constant characterizing the channel sensitivity. To speed up the data acquisition in monitoring cerebral hemodynamic responses, TPSF measurements were performed differently in the rest and task periods. During the resting-state measurement (typically 30 sec relaxation), full-length TPSF signals with 13 delays ($k=1,2,\ldots,13$) were acquired for fitting background optical properties. In the task period, however, the TPSFs were sampled at only three delays: $k=k_{p},k_{p}+1,k_{p}+2.$ Here $k_{p}$ was the delay point where the signal reached its peak value. Both non-linear fitting algorithms and analytical solutions [10] were employed to derive the HbO, HbR levels and their dynamic changes from the time-domain measurements.


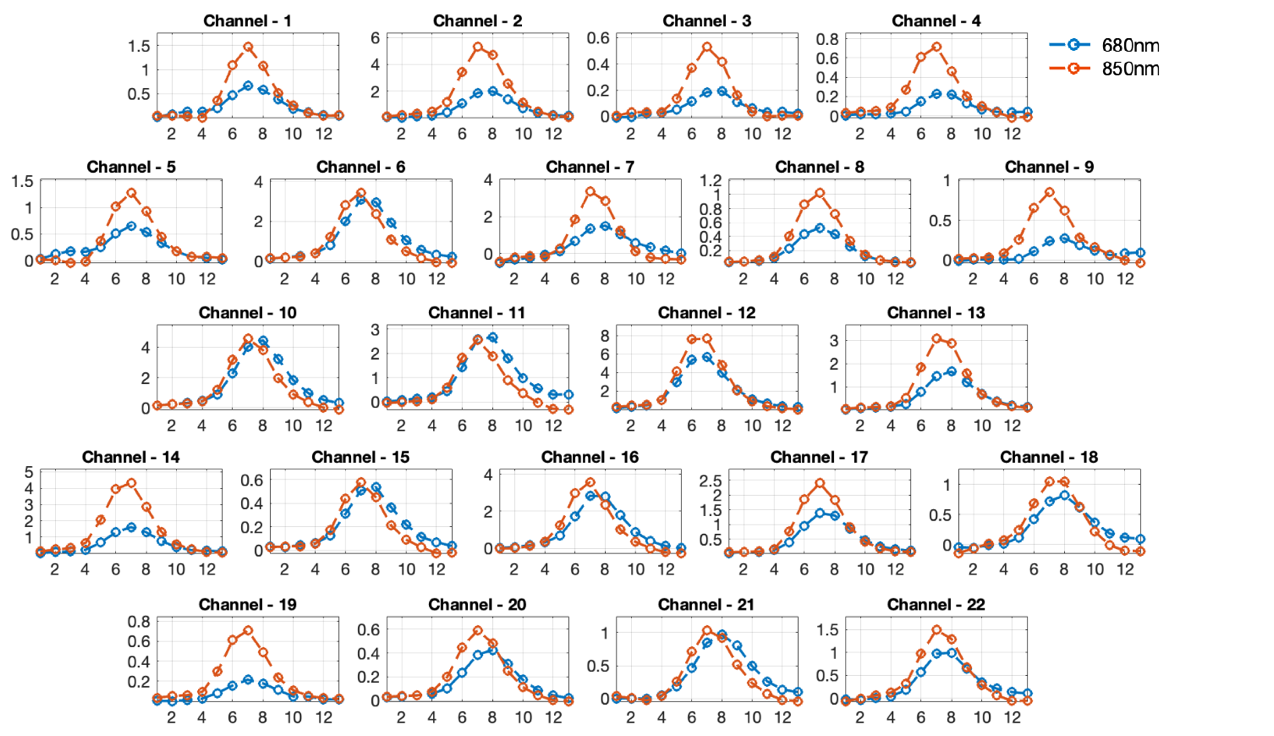


1. Baseline TPSF signals with 13 delays and two wavelengths measured from the PFC region during resting state; Blue: 680 nm, Red: 850 nm. Horizontal axes: delay indices; Vertical axes: signal intensity.
2. **Estimation of hemoglobin concentration from the time-domain fNIRS measurements**

There were two analysis methods employed to convert TPSF signals into hemoglobin-relevant physiological parameters. (1) The Modified Beer-Lambert Law (MBLL, Eq. (1) ) was applied to a single measurement point (i.e., the fluence rate at a specific time delay) in TPSF to obtain relative changes in oxygenated hemoglobin (∆HbO), deoxygenated hemoglobin (∆HbR), and total hemoglobin (∆HbT) [16]. (2) Based on the diffusion equation for a homogeneous infinite model and the optical absorption spectra of hemoglobins, the absolute values of HbO, HbR, and HbT concentrations could be derived from two measurement points in TPSFs. The relevant calculations were provided by Eqs. (2)(3).


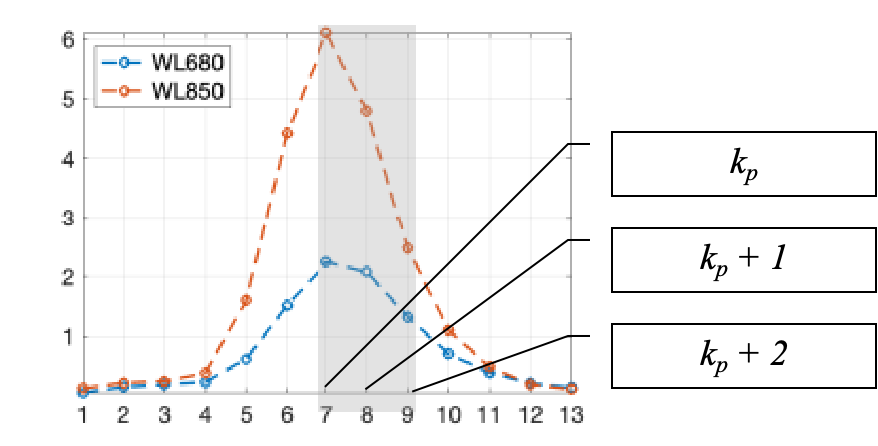


1. The TPSF signals measured from the forehead; blue and red lines denote two wavelengths, 680 nm and 850 nm, respectively. The fluence rates in the latter part of TPSF are correlated to the hemoglobin concentration in the tissue.
2. **Modified Beer–Lambert Law**

The time-domain fNIRS signals can be analyzed on the basis of Modified Beer–Lambert Law (MBLL), which is expressed by the formula:

| $\Delta OD\left( \lambda\right)= \Delta\left[ -log\left( \Phi\left( \lambda,t_{k} \right) \right) \right]=\sum_{i} \varepsilon_{i}\left( \lambda\right)\nu t_{k}\times{\Delta C}_{i}$ | (1) |
| --- | --- |

where OD represents the measured attenuation, so called ‘optical density’, $\Phi$ is the fluence rate at a specific wavelength ($\lambda=680 nm and 850 nm$) and time $t_{k}$ ($k=k_{p},k_{p}+1,k_{p}+2$), *ε* is the extinction coefficient [14], *ν* is the velocity of light propagating in tissue, *C* is the concentration of the specific chromophore (hemoglobin is the element of interest here, *i* $=$ HbO, HbR). This calculation to relative changes of HbO and HbR (i.e., $\Delta_{HbO}$ and $\Delta_{HbR}$) is sensitive to changes in optode-scalp coupling and surface interferences [15][19].

1. **Diffusion equation (DE) model**

Previous studies [11][12] reported that the logarithmic slopes of Temporal Point Spread Function (TPSF) distribution in the decaying period were proportional to hemoglobin concentrations in biological tissues by time-domain measurements, which detect the diffusely reflected light at some distance from the source as a function of time (see Figure S4). An analytical solution of the diffusion equation in an infinite homogeneous medium (Green’s function) to fast infer the absorption coefficient *μ_a_* correlated to hemoglobin (Hb) concentration *C* can be described as followings:

| $\Phi\left( \lambda,\boldsymbol{r},t_{k} \right)=\frac{\nu}{\left( 4\pi D\nu t_{k} \right)^{3/2}}\times exp\left( -\frac{\left\vert\boldsymbol{r} \right\vert^{2}}{4D\nu t_{k}} \right)\times exp\left( -\mu_{a}\left( \lambda\right)\nu t_{k} \right)$ | (2) |
| --- | --- |
| $\mu_{a}\left( \lambda\right)=2.303\sum_{i=HbO,HbR} \varepsilon_{i}\left( \lambda\right)C_{i}$ | (3) |

where $\Phi$ is the fluence rate corresponding to the amplitudes of TPSF, which is related with the used light wavelength ($\lambda=680 nm and 850 nm$), $\boldsymbol{r}$ is the distance from a point impulse source located at the origin, and *k* is a selected time delay of TPSF, for each wavelength, at least two out of three time delays (e.g., $k_{p}$ and $(k_{p}+1)$ vs. $(k_{p}+1)$ and $(k_{p}+2)$ are used to fit the fluence rate to the Green’s function.); The diffusion coefficient *D* can be estimated from a baseline full time spectrum measured during the resting state.

Since the measured TPSF signals could be resolved to $\mu_{a}\left( \lambda\right)$ by the above analytical solution Eq. (2), with the knowledge of the extinction coefficients of HbO and HbR [14], it is able to estimate the levels of HbO and HbR in the tissue by applying the Eq. (3). Solving the simultaneous equations for the two wavelengths used in the TD-fNIRS system provides $C_{HbO}$, $C_{HbR}$, and $C_{HbT}=C_{HbO}+C_{HbR}$. Advantages of DE model [9][10]: (1) The DE-based solutions are less sensitive to motion artefacts, varying coupling conditions, and surface interferences; (2) It is possible to emphasize early or late arriving photons in time-delay selection to differentiate responses in shallow and deep regions.

1. **TD-fNIRS data processing and classification**

The flowchart of TD-fNIRS data processing is shown in Figure S5. First, TPSF signals with a peak intensity falling out of the range [0.5, 6] were identified as corrupted channels (either too weak or saturated) and excluded from further activation analysis. Second, as the TD-fNIRS signals were oversampled and the analysis was aimed at extracting features that were relevant to relatively slow, tasked-evoked hemodynamic responses, a low-pass moving average filter with a window size of 10 sec was utilized to remove cardiac pulsation (0.8~1.6 Hz), respiration (0.2~0.6 Hz), and motion artifacts (> 1 Hz) [15].

The analysis primarily concentrated on the HbO response, as it is regarded as a more direct indicator of task-related cortical activation compared to other signals. This is supported by its strong correlation with the blood oxygenation level-dependent (BOLD) signal observed in fMRI studies [17] and the findings of relevant animal research [18]. In addition to analyzing the $\Delta_{HbO}$ data obtained by the MBLL, which is commonly used in fNIRS studies, we also explored the task-rest oxygen-hemoglobin ratio $R_{HbO}$ provided exclusively by the TD-fNIRS instrument to study hemodynamic responses. The oxygen-hemoglobin change $C_{HbO}^{S}$ during the stimulus-task period was divided by its concentration level $C_{HbO}^{P}$ in the pre-task resting state to derive the task-rest HbO ratio, i.e., $R_{HbO}=ln\left( {C_{HbO}^{S}}/{C_{HbO}^{P}} \right)$. Calculating the natural logarithm of the ratio of task and rest state HbO concentrations is a dimensionless measure that enhances the detection of physiological response intensity in brain activity resulting from task stimulation, while reducing individual variability in systemic hemoglobin concentration. Additionally, for the computation of activation features, the HbO data including 10 sec pre-task, task duration, and 50 sec post-task were extracted. Subsequently, the time-series $\Delta_{HbO}$ or $R_{HbO}$ data were linearly fitted using the 10-sec pre-baseline and the last 10-sec post-baseline (see Figure S1).

In the final stage of analysis, time-series HbO data from valid channels were classified into three hemodynamic patterns: activated, suppressive, and channel-wise averaged $\Delta_{HbO}$ and $R_{HbO}$ responses. This allowed us to implement feature extraction for visualized data analysis and to investigate the association between stimulated responses and depression-relevant function in the PFC region, the process details were shown in Figure S6.


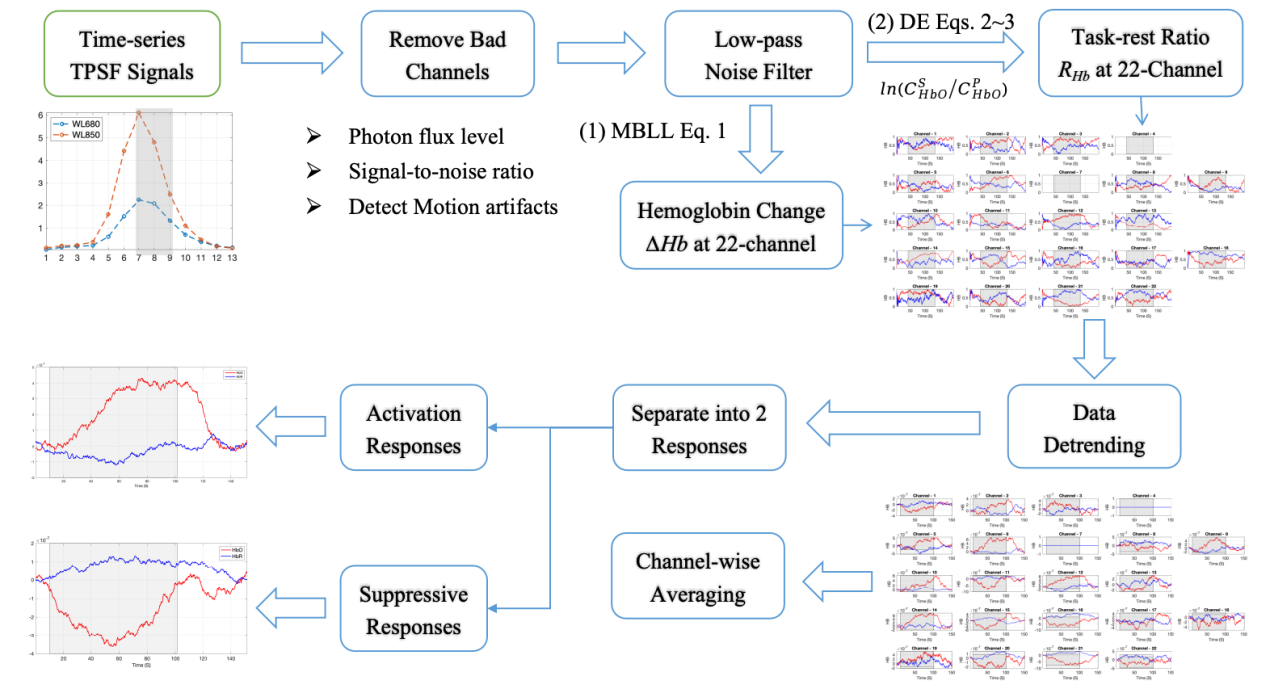


1. Data processing steps for converting raw TPSF signals into time-series HbO responses. The equations were provided in the Supplementary.


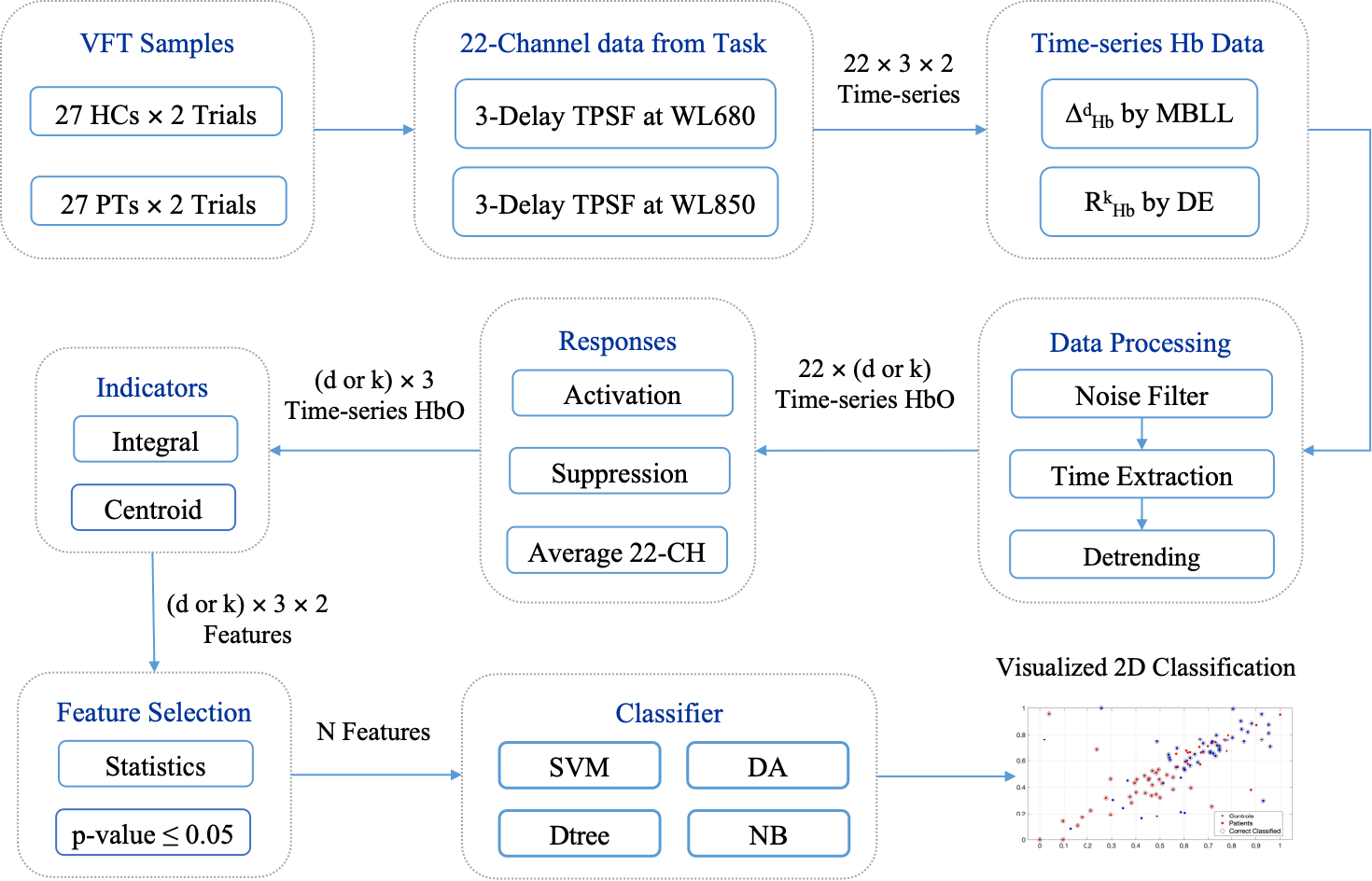


1. Data analysis processes, including data transformation, feature selection and visualized classification.

**Results:**

1. **Analysis results with the relative change of oxygenated hemoglobin (**$\boldsymbol{\Delta}_{\mathbf{HbO}}$**) obtained by MBLL**


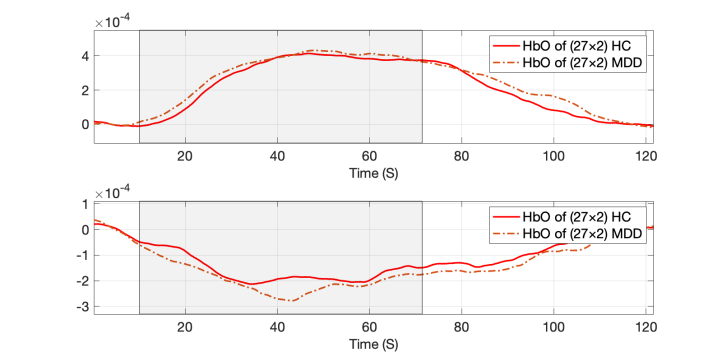

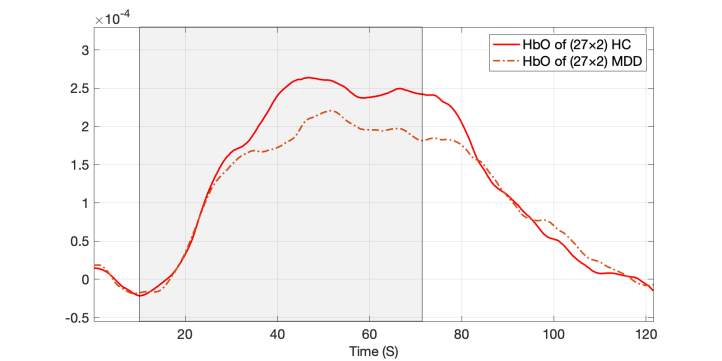


(a1) (a2)


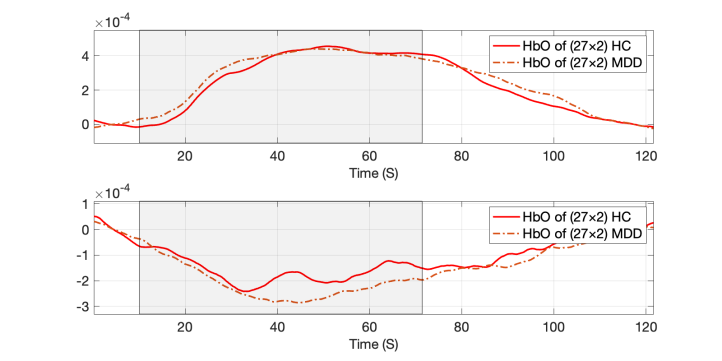

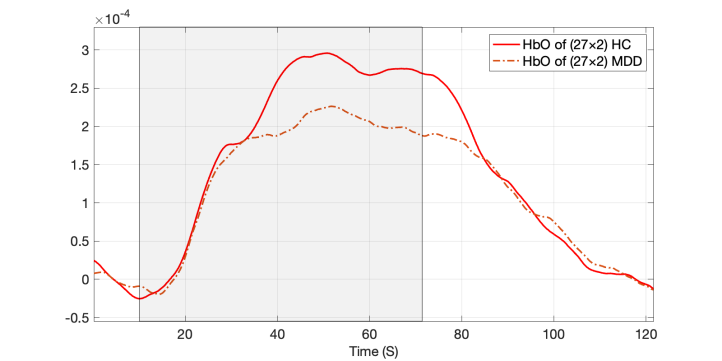


(b1) (b2)


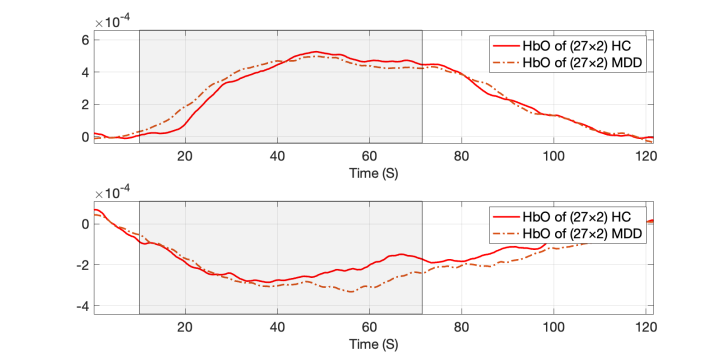

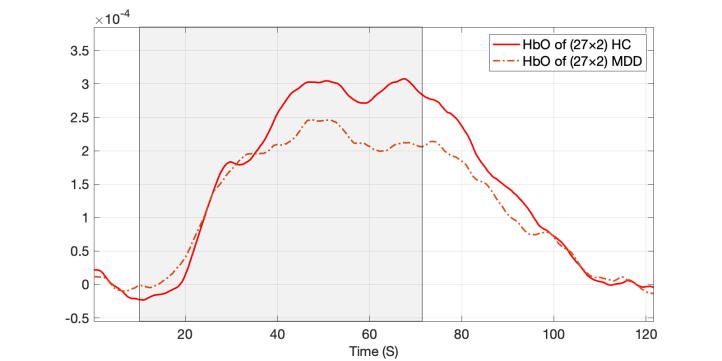


(c1) (c2)

1. Comparison of average oxy-hemoglobin responses between HC and MDD during VFT (gray area). The HbO change $\Delta_{HbO}$ (unit: moles/liter) obtained by MBLL with the (a) 1^st^, (b) 2^nd^ and (c) 3^rd^ delay of TPSF signals. Left: activated and suppressive responses; Right: channel-wise averaged response.
2. Classification accuracy of 2D and 4D features from the $\Delta_{HbO}$ response.

| **Features** | **Linear SVM** | **Discriminant Analysis** | **Decision Trees** | **Naive Bayes** |
| --- | --- | --- | --- | --- |
| F6 & F8 | 62.0% | 64.8% | 64.8% | 63.0% |
| F6 & F9 | 62.0% | 63.0% | 64.8% | 64.8% |
| F6 & F12 | 57.4% | 59.3% | 60.2% | 57.4% |
| F8 & F9 | 55.6% | 61.1% | 66.7% | 58.3% |
| F8 & F12 | 57.4% | 61.1% | 67.6% | 66.7% |
| F9 & F12 | 62.0% | 63.0% | 63.0% | 62.0% |
| 4D Features | 60.2% | 62.0% | 64.8% | 62.0% |

**Note:**

F6: Centroid of channel-averaging at delay 1 of TPSF

F8: Centroid of Activation at delay 2 of TPSF

F9: Integral of Suppression at delay 2 of TPSF

F12: Centroid of channel-averaging at delay 2 of TPSF

4D Features: F6, F8, F9, and F12

1. Five-fold cross-validation accuracy (Mean ± Std.) of 2D and 4D Features from the $\Delta_{HbO}$ response.

| **Features** | **Linear SVM** | **Discriminant Analysis** | **Decision Trees** | **Naive Bayes** |
| --- | --- | --- | --- | --- |
| F6 & F8 | 62.9% ± 7.6% | 62.0% ± 11.9% | 60.0% ± 8.6% | 63.9% ± 5.2% |
| F6 & F9 | 61.1% ± 6.0% | 60.2% ± 7.6% | 50.1% ± 4.9% | 54.8% ± 6.5% |
| F6 & F12 | 57.4% ± 8.2% | 58.3% ± 4.1% | 50.9% ± 4.7% | 49.0% ± 6.2% |
| F8 & F9 | 52.0% ± 11.1% | 60.1% ± 7.6% | 50.0% ± 4.4% | 55.4% ± 10.6% |
| F8 & F12 | 59.5% ± 15.7% | 60.1% ± 8.2% | 48.1% ± 7.1% | 56.5% ± 7.1% |
| F9 & F12 | 59.4% ± 8.7% | 61.1% ± 5.4% | 49.9% ± 7.2% | 50.8% ± 9.4% |
| 4D Features | 59.3% ± 9.8% | 60.3% ± 8.5% | 48.1% ± 11.6% | 57.3% ± 8.4% |

1. **Analysis results with the task-rest ratio of oxygenated hemoglobin (**$\boldsymbol{R}_{\boldsymbol{HbO}}$**) derived from TD-fNIRS measurements**


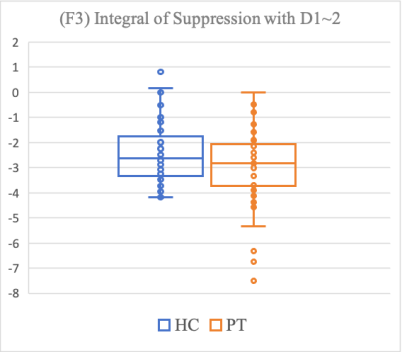

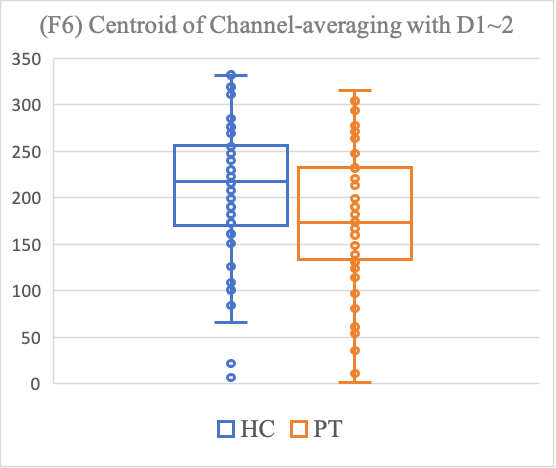

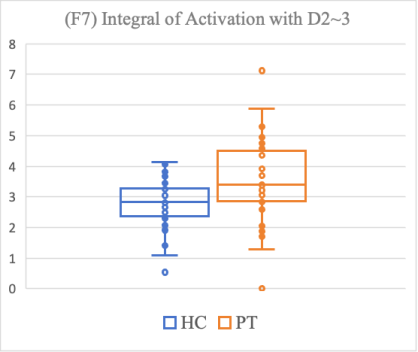


(a) (b) (c)


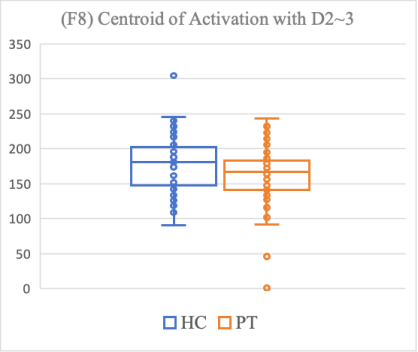

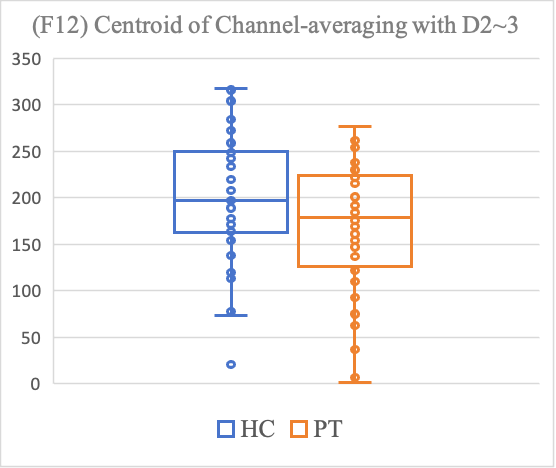


(d) (e)

1. Box-plot comparisons of the five $R_{HbO}$ features with significant difference (p-value ≤ 0.05) between patients with MDD (PT) and healthy controls (HC).

**
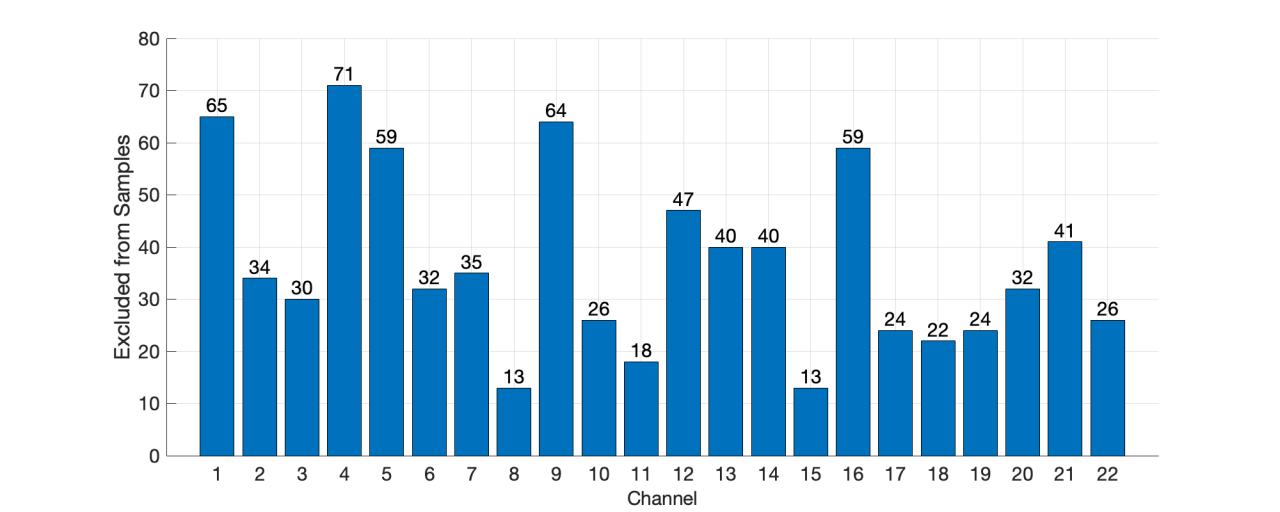
**

1. Channel rejection across 108 trials (54 participants × 2 VFT sessions) using 22-channel TD-fNIRS: 7.5 ± 4.1 channels/trial excluded from analysis.
2. Classification accuracy of 2D and 5D features from the $R_{HbO}$ response.

| **Features** | **Linear SVM** | **Discriminant Analysis** | **Decision Trees** | **Naive Bayes** |
| --- | --- | --- | --- | --- |
| F3 & F6 | 60.2% | 58.3% | 65.7% | 66.7% |
| F3 & **F7** | 66.7% | 63.0% | 71.3% | 70.4% |
| F3 & F8 | 57.4% | 57.4% | 66.7% | 65.7% |
| F3 & F12 | 60.2% | 59.3% | 66.7% | 62.0% |
| F6 & **F7** | 68.5% | 66.7% | 72.2% | 71.3% |
| F6 & F8 | 65.7% | 63.9% | 69.4% | 66.7% |
| F6 & F12 | 62.0% | 61.1% | 71.3% | 71.3% |
| **F7** & F8 | 74.1% | 73.1% | 72.2% | 75.0% |
| **F7** & F12 | 74.1% | 74.1% | 72.2% | 72.2% |
| F8 & F12 | 60.2% | 60.2% | 63.0% | 61.1% |
| 5D Features | 77.8% | 78.7% | 77.8% | 82.4% |

**Note:**

F3: Integral of suppression at delays 1~2 of TPSF

F6: Centroid of channel-averaging at delays 1~2 of TPSF

**F7: Integral of Activation at delays 2~3 of TPSF**

F8: Centroid of Activation at delays 2~3 of TPSF

F12: Centroid of channel-averaging at delays 2~3 of TPSF

5D Features: F3, F6, F7, F8, and F12

1. Five-fold cross-validation accuracy (Mean ± Std.) of 2D and 5D Features from the $R_{HbO}$ response.

| **Features** | **Linear SVM** | **Discriminant Analysis** | **Decision Trees** | **Naive Bayes** |
| --- | --- | --- | --- | --- |
| F3 & F6 | 57.5% ± 7.4% | 59.2% ± 9.7% | 61.0% ± 7.4% | 65.7% ± 4.7% |
| F3 & **F7** | 66.7% ± 4.9% | 63.9% ± 9.9% | 65.8% ± 7.0% | 61.1% ± 7.2% |
| F3 & F8 | 55.8% ± 15.0% | 53.6% ± 7.1% | 51.9% ± 9.8% | 56.4% ± 5.9% |
| F3 & F12 | 58.4% ± 8.1% | 56.4% ± 7.8% | 50.0% ± 5.4% | 52.7% ± 3.0% |
| F6 & **F7** | 66.8% ± 12.3% | 69.5% ± 10.7% | 64.8% ± 8.3% | 66.7% ± 11.6% |
| F6 & F8 | 64.8% ± 10.8% | 59.3% ± 7.5% | 63.0% ± 6.4% | 58.2% ± 6.5% |
| F6 & F12 | 60.1% ± 7.5% | 60.3% ± 8.1% | 55.5% ± 7.6% | 60.3% ± 11.6% |
| **F7** & F8 | 73.2% ± 5.8% | 75.1% ± 6.6% | 63.9% ± 6.5% | 62.9% ± 13.3% |
| **F7** & F12 | 68.7% ± 9.4% | 73.2% ± 3.7% | 60.2% ± 6.9% | 63.9% ± 7.4% |
| F8 & F12 | 59.2% ± 6.7% | 54.7% ± 8.3% | 49.0% ± 7.4% | 51.8% ± 9.9% |
| 5D Features | 71.3% ± 8.1% | 78.7% ± 12.5% | 62.0% ± 15.0% | 74.8% ± 8.9% |

# References

1. Kakimoto Y, Nishimura Y, Hara N, Okada M, Tanii H, Okazaki Y. Intrasubject reproducibility of prefrontal cortex activities during a verbal fluency task over two repeated sessions using multi-channel near-infrared spectroscopy. Psychiatry and Clinical Neurosciences. 2009;63(4):491–9.
2. Schecklmann M, Ehlis AC, Plichta MM, Fallgatter AJ. Functional near-infrared spectroscopy: A long-term reliable tool for measuring brain activity during verbal fluency. NeuroImage. 2008 Oct 15;43(1):147–55.
3. Lang X, Wen D, Li Q, Yin Q, Wang M, Xu Y. fNIRS Evaluation of Frontal and Temporal Cortex Activation by Verbal Fluency Task and High-Level Cognition Task for Detecting Anxiety and Depression. Front Psychiatry. 2021;12:690121.
4. Pu S, Nakagome K, Miura A, Iwata M, Nagata I, Kaneko K. Associations between depressive symptoms and fronto-temporal activities during a verbal fluency task in patients with schizophrenia. Sci Rep. 2016 July 28;6(1):30685.
5. Chen NG, Zhu Q. Time-resolved optical measurements with spread spectrum excitation. Opt Lett, OL. 2002 Oct 15;27(20):1806–8.
6. Chen NG, Zhu Q. Time-resolved diffusive optical imaging using pseudo-random bit sequences. Opt Express, OE. 2003 Dec 15;11(25):3445–54.
7. Zhang Q, Chen L, Chen N. Pseudo-random single photon counting: a high-speed implementation. Biomed Opt Express, BOE. 2010 Aug 2;1(1):41–6.
8. Okamoto M, Dan H, Sakamoto K, Takeo K, Shimizu K, Kohno S, et al. Three-dimensional probabilistic anatomical cranio-cerebral correlation via the international 10–20 system oriented for transcranial functional brain mapping. NeuroImage. 2004 Jan 1;21(1):99–111.
9. Mehta KB, Hasnain A, Zhou X, Luo J, Penney TB, Chen N. Spread spectrum time-resolved diffuse optical measurement system for enhanced sensitivity in detecting human brain activity. JBO. 2017 Apr;22(4):045005.
10. Yamada Y, Suzuki H, Yamashita Y. Time-Domain Near-Infrared Spectroscopy and Imaging: A Review. Applied Sciences. 2019 Jan;9(6):1127.
11. Chance B, Nioka S, Kent J, McCully K, Fountain M, Greenfeld R, et al. Time-resolved spectroscopy of hemoglobin and myoglobin in resting and ischemic muscle. Analytical Biochemistry. 1988 Nov 1;174(2):698–707.
12. Riley J, Hassan M, Chernomordik V, Gandjbakhche A. Choice of data types in time resolved fluorescence enhanced diffuse optical tomography. Medical Physics. 2007;34(12):4890–900.
13. Cuccia DJ, Abookasis D, Frostig RD, Tromberg BJ. Quantitative In Vivo Imaging of Tissue Absorption, Scattering, and Hemoglobin Concentration in Rat Cortex Using Spatially Modulated Structured Light. In: Frostig RD, editor. In Vivo Optical Imaging of Brain Function [Internet]. 2nd edn Boca Raton (FL): CRC Press/Taylor & Francis; 2009.
14. Prahl SA. Tabulated molar extinction coefficient for hemoglobin in water. http://omlc.ogi.edu/spectra/hemoglobin/summary.html. 1999.
15. Zhang F, Cheong D, Khan AF, Chen Y, Ding L, Yuan H. Correcting physiological noise in whole-head functional near-infrared spectroscopy. Journal of Neuroscience Methods. 2021 Aug 1;360:109262.
16. Kocsis L, Herman P, Eke A. The modified Beer–Lambert law revisited. Phys Med Biol. 2006 Feb;51(5):N91.
17. Strangman G, Culver JP, Thompson JH, Boas DA. A Quantitative Comparison of Simultaneous BOLD fMRI and NIRS Recordings during Functional Brain Activation. NeuroImage. 2002 Oct 1;17(2):719–31.
18. Hoshi Y, Kobayashi N, Tamura M. Interpretation of near-infrared spectroscopy signals: a study with a newly developed perfused rat brain model. Journal of Applied Physiology. 2001 May;90(5):1657–62.
19. Zhou X, Sobczak G, McKay CM, Litovsky RY. Comparing fNIRS signal qualities between approaches with and without short channels. PLOS ONE. 2020 Dec 23;15(12):e0244186.
